# Growing debt burden in low- and middle-income countries during COVID-19 may constrain health financing

**DOI:** 10.1080/16549716.2022.2072461

**Published:** 2022-06-22

**Authors:** Frederik Federspiel, Josephine Borghi, Melisa Martinez-Alvarez

**Affiliations:** a Department of Global Health and Development, London School of Hygiene and Tropical Medicine, London, UK; b MRC Unit the Gambia at the London School of Hygiene and Tropical Medicine, Banjul, The Gambia

**Keywords:** Debt, development loans, COVID-19, health financing

## Abstract

Debt burdens are growing steadily in Low- and Middle-Income Countries (LMICs), compounded by the COVID-19 economic recession, threatening to crowd out essential health spending. In 2019, 54 LMICs spent more on servicing their debt to foreign creditors than on financing their health services. While development loans may have positive effects on population health, the ensuing debt servicing requirements may have detrimental effects on health through constrained fiscal space for government health spending. However, the existing evidence is inadequate for an understanding of whether, and if so how and under what circumstances, debt may constrain government health spending. We call for more research on the impacts of debt on health financing and call on creditors and borrowers to carefully consider the potential impacts of lending on borrower countries’ ability to finance their health services.

Low- and Middle-Income Country (LMIC) governments have become increasingly indebted over the past decade (Figure 1) [1]. Concurrently, debt repayment levels have grown steadily, channelling funds out of LMIC government budgets to public and private creditors in High-Income Countries (HICs) (Figure 2) [1]. The number of LMICs that spend more on debt servicing than health has increased substantially from 33 in 2010 to 54 in 2019 (Figures 3 and 4) [1,2]. In 2019, four countries, Angola, Benin, Cameroon and the Republic of the Congo, spent at least five times more on external debt servicing than they spent on health. While there is no evidence of a causal link between increased debt servicing and lower health spending, reductions in the public budget due to debt servicing will likely have implications for sectoral budget allocations, including health. Government health spending is essential for the functioning of all publicly funded health services from prevention to cure. The deeper the levels of poverty in a population, the less people can afford or have access to private health services, and government health financing is crucial to achieve Universal Health Coverage (UHC), serving all of the population, reducing health inequalities, and protecting people from financial risk when using health services [3,4]. The growing debt burden in LMICs seen in Figures 1–3 therefore gives cause for concern of constrained fiscal space for health and the implications hereof. Indeed, based on observational data similar to that presented in Figures 2–4, the Jubilee Debt Campaign has brought attention to the countries spending more on debt than health in the beginning of the pandemic and called for debt service cancellation for the year 2020 [5].

**Figure 1.**
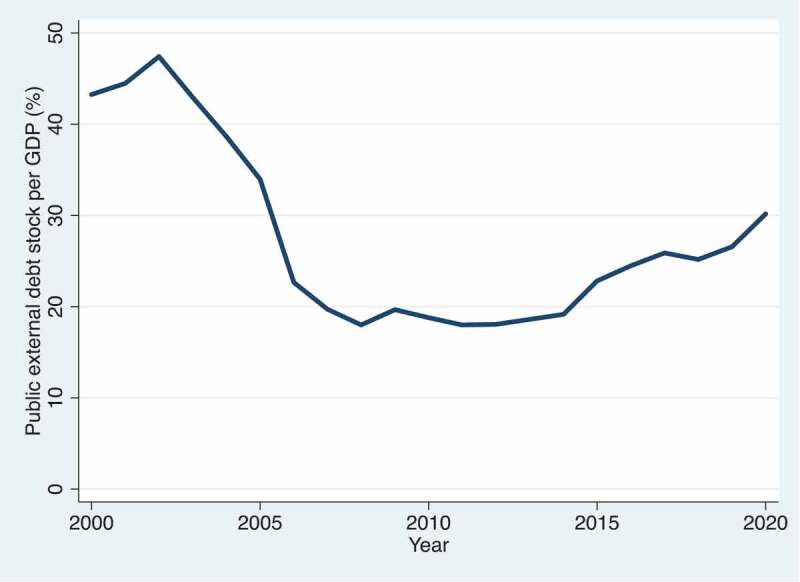
Public external debt stock as a proportion of Gross Domestic Product (GDP) in Low- and Middle-Income Countries (LMICs) from 2000–2020 [1].

**Figure 2.**
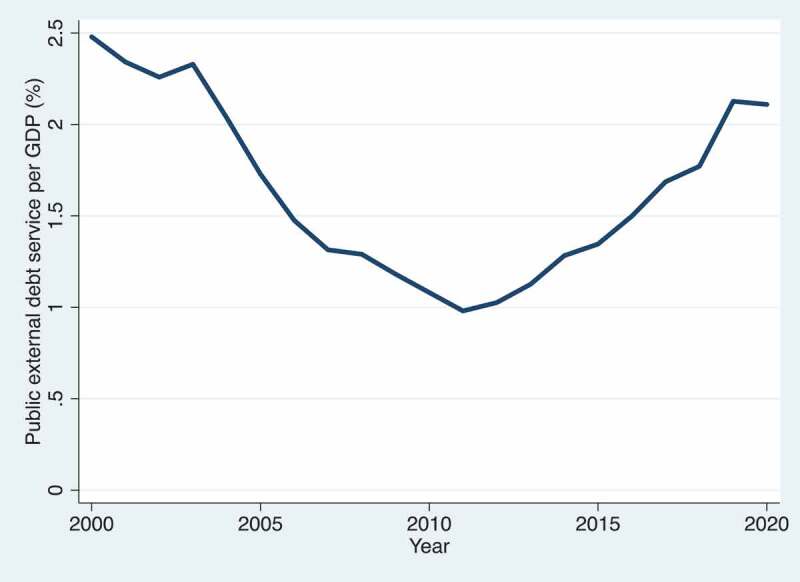
Public external debt service as a proportion of Gross Domestic Product (GDP) in Low- and Middle-Income Countries (LMICs) from 2000–2020 [1].

**Figure 3.**
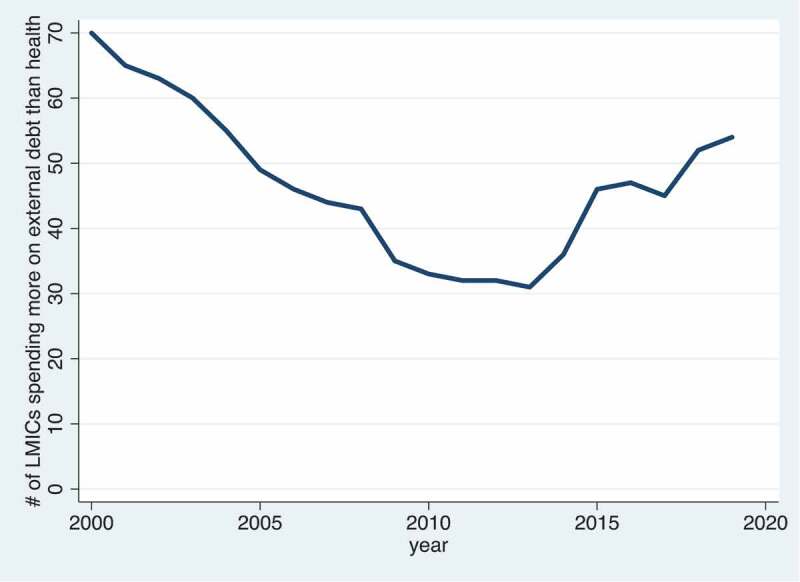
Number of Low- and Middle-Income Countries (LMICs) that spent more on public external debt servicing than health, 2000–2019 [1,2].

**Figure 4.**
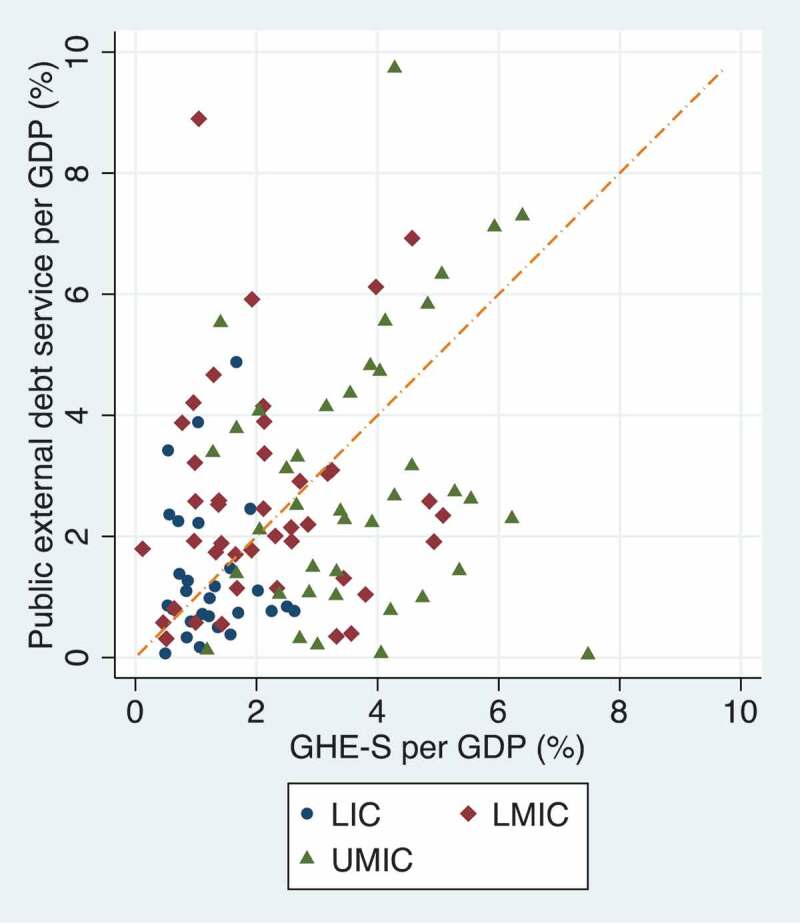
Public external debt service and government health expenditure from domestic revenue (GHE-S) as fractions of Gross Domestic Product (GDP) (%) among Low-and Middle-Income Countries (LMICs) in 2019 [1,2]. The diagonal line indicates equal spending on debt servicing and health. Countries above the line spend more on external debt than on health and vice versa.

## COVID-19 has led to more development lending which may increase debt levels

COVID-19 has triggered the largest economic recession since World War II [6]. This has eroded the tax base in many LMICs, contracting government revenue and leaving a rift between countries expected to recover economically and others experiencing lasting damage to government revenue [6]. Early WHO estimates from countries with available national health accounts data for 2020 – mainly HICs – indicate public health spending increases, which may reflect increased funds to help manage the pandemic [7]. This may, however, not be the case for LMICs operating under severe public resource constraints, and where government revenue does not show signs of recovery after the initial shock [6]. In such settings, COVID-19 may take up already scarce health resources for its prevention and management, and studies are ongoing to assess the impacts of emergency expenditure reallocation to manage COVID-19 on the rest of the health budget in Pakistan and South Africa [8]. COVID-19 also adds to the health burden through worsened mental [9] and maternal health [10], amongst others, increasing the need for health financing.

In addition to these impacts, COVID-19 also appears to have affected the levels of development assistance committed by donors and the balance between loans and grants. Total aid commitments made in 2020 increased by 19% in real terms compared to 2019 [11]. This increase was made up almost entirely by development loan commitments, presumably to help countries cope with the impacts of the pandemic, while grant commitments stayed at approximately the same level [11]. While increased donor commitments is positive, it will be important to monitor first of all disbursement rates of these commitments over the following years, but also if a new normal will be established, where development loans are a more prominent mode of providing development assistance, which will in turn further increase debt levels.

## What do we know about the relationship between debt and health expenditure?

Our empirical understanding of the full impacts of development lending, the ensuing debt stock and its servicing on health financing in borrower countries is, however, limited and conflicting. Three studies of countries in Africa (using data from 1975 to 1994) [12], Latin America (1985–2003) [13] and South and Southeast Asia (1980–2010) [14] have found a higher debt burden to be associated with less government health spending or less overall social spending, while a larger study of 120 LMICs found the opposite (1995–2010) [15]. Other research has identified constraining effects on government health spending of IMF loan conditionalities (e.g. [16,17]). The identified empirical studies [12–17] are all quantitative multi-country studies. We therefore have a limited understanding of which particular social, economic and political country characteristics and which lending arrangements constrain health spending or support it. Neither do we fully understand whether or how debt servicing affects equity by impacting the distribution of health financing in a given country and resultingly health service access for different population groups. Such in-depth analysis may be necessary to be able to provide relevant policy recommendations to creditors and borrowers.

Another important consideration is that the debt of LMICs originates from various sources, with much coming from concessional Official Development Assistance (ODA) loans. These may have gone to the health sector in the first place, such as for constructing health centres, and may thus have a beneficial effect on population health. The proportion of ODA loan disbursements going to health is however rather small, at less than 4% of all ODA loans to LMICs in 2019 [11]. Development lending to other sectors such as the productive, economic and infrastructure sectors may also have effects on health, which may be beneficial, for example, through improved food security or improved financial or geographical access to health services, or harmful, for example, through pollution or increased availability of health-harmful commodities. Additionally, development lending to these sectors may lead to economic growth which, if taxed and partly allocated to the health sector, may expand government health spending. These considerations and the data presented in Figures 1–4 give rise to several questions: Does more public external debt servicing lead to less government health spending, and vice versa? Do the positive effects of development lending on health financing outweigh the negative effects, if any, of debt repayments? What are the effects of development loans and the associated debt burden on equity in health financing in borrower countries?

In the 2000s, a steady decline in debt repayments occurred after the Heavily Indebted Poor Countries (HIPC) initiative and the Multilateral Debt Relief Initiative (MDRI) were launched in 1996 and 2005, respectively, offering debt relief to 39 heavily indebted LMICs [18,19]. While some researchers have discussed the potential of these initiatives to free up funds for health financing [20,21], to the authors’ knowledge, no statistical testing of the actual health financing impacts of this natural policy experiment have been performed, and neither have dedicated case-country policy analyses. Nor are we aware of any analyses of the real health financing impacts of the recent G20 Debt Service Suspension Initiative, temporarily delaying debt servicing requirements for 2020 and 2021 for participating countries [22]. As such, it is not known whether and under what circumstances the assumption of decreased debt servicing freeing up fiscal space actually translates into more health spending.

## Recommendations

Figures 1 and 2 show concerning trends in the debt obligations of LMICs, while Figures 3 and 4 illustrate the level of debt servicing relative to health expenditure in LMICs. While COVID-19 has shown the world the necessity and importance of health spending, it has also eroded government revenue in many countries and has elicited an increase in development lending. These events prompt us to call for more empirical, peer-reviewed research analysing the following questions:

First, does increased external debt servicing result in lower government health spending? If so, under what circumstances? Conversely, does increased fiscal space generated from decreased debt servicing lead to increased health spending? If so, under what circumstances?

Second, do development loans to health and other sectors have a net positive effect on health financing in the long run, when debt repayments are taken into account? What are some individual country experiences in regard to this question?

Third, what are the implications of development loans and ensuing external debt for equity in health financing in borrower countries?

We believe a range of different methodological approaches from the social and public health sciences, both quantitative and qualitative, will be necessary to address these questions. The answers may vary greatly between countries, and in-depth case country studies may be necessary to address these questions in sufficient detail and understand individual country realities.

We ask for creditors to be keenly aware of the illustrated growth in external debt obligations of borrower countries, and to carefully consider the potential short- and long-term impacts of lending on the ability of these countries to finance their health services. The growing debt burdens in LMICs may mean that debt relief will at some point again have to be considered to ensure continued sustainable development in borrower countries. Debt-to-health swaps, where funds freed up from debt relief are earmarked for health purposes, are one way to ensure that debt relief translates into increased health spending (alongside debt-to-education swaps, debt-to-environment swaps, etc.) [23].

For borrower countries, we ask policy makers to prioritize social sectors and safeguard these from budget cuts when debt repayments have to be made. We ask them to carefully consider the short- and long-term social and environmental implications of taking up a development loan, and to exercise strong vigilance about the type of creditor and lending arrangements engaged in, to avoid a disproportional burden of repayment being passed on to the next generation, with ensuing possible implications for the fiscal space for social spending.

## Paper context

Existing evidence on the associations between public external debt obligations in Low- and Middle-Income Countries (LMICs) and health spending is scarce and conflicting. Meanwhile, the debt burdens of LMICs are growing steadily, potentially constraining essential health spending. This short communication calls to action both creditors and borrowers to carefully consider the health financing impacts of lending and asks the research community to investigate the effects of debt on health financing in LMICs.
